# The Causes of Death in Five Cases in a Colliery Explosion

**Published:** 1897-09

**Authors:** Trafford Mitchell


### THE CAUSES OF DEATH IN FIVE CASES IN A COLLIERY EXPLOSION.

BY

TRAFFORD MITCHELL, M.D. Glasg., D.P.H. Camb.

---

ON the morning of 5th January, 1897, an explosion occurred in the Broadoak Colliery, Loughor, Glamorganshire, by which five out of the seven men at that time underground were killed. Fortunately, the explosion took place after most of the night men had left the work and before the colliers had descended.

In view of the interest excited by Haldane's *Report on the Causes of Death in Colliery Explosions*, and the prominent part he assigns to carbon monoxide in the production of the fatal results, H.M. Inspector of Mines (Mr. J. T. Robson) directed me to make a thorough external examination of the bodies, with a view to discovering the probable cause of death in each case. With the assistance of Dr. A. C. Davies, I did so eleven hours after the explosion, and the following is a brief statement of the appearances noted :—

Bodies Nos. 1, 2 and 3 exhibited superficial burns of the exposed surfaces, with detachment of the cuticle, except in the case of the palmar surfaces. The hair of the head and face was in each case singed, but the clothes were uninjured.

Bodies Nos. 4 and 5 showed no evidence of burning or violence.

Body No. 1 was markedly differentiated from the other four by the fact that cadaveric lividity was intense, that the insides of the lips were almost coal black, and that the eyes were suffused with dark blood.

Bodies Nos. 2 and 3 were distinguished by a pale, bloodless condition of the lips, eyes congested and pink, and the dependent parts bright pink. In No. 2 both hands were clenched; in No. 3 only the right one.

Bodies Nos. 4 and 5 showed the same condition of the eyes as Nos. 2 and 3; but the lips were of the same colour as in life, the countenance abnormally florid, and the expression tranquil, whilst the dependent parts were brightly pink and the other surfaces less markedly so. No. 4 grasped his lamp, whilst No. 5 held his cap firmly in his right hand.

The first body was evidently that of one who had been asphyxiated through deficiency of oxygen and excess of carbon dioxide. That this man's safety lamp was open at the time of the explosion was clearly demonstrated at the inquest. In his endeavour to escape he had run a distance of twenty yards from the spot at which the two halves of his lamp were found.

The fourth body was unmistakably that of a man poisoned by carbon monoxide. To confirm my own conclusion, I requested Mr. Seyler, the public analyst, to make a spectroscopic examination of a small quantity of blood removed from this body on the afternoon of the 6th January, and he found the spectroscopic reaction for carbon monoxide very marked. Ere succumbing this man had run a distance of over 100 yards, and the body was discovered by the exploring party following his footsteps.

Body No. 5 was equally unmistakably, with No. 4, a case of carbon monoxide poisoning. He also had tried to escape, but had not succeeded in running far.

Nos. 2 and 3 at the time of the explosion were working at a distance of about sixty yards from the point where the explosion commenced and in its direct line. They had made no effort to escape, and were found lying beside the tools with which they had been engaged filling rubbish into a small tram, to which a horse was attached. Both these men were apparently in the first place rendered insensible by sudden and intense shock, upon which supervened the action of carbon monoxide. The effect upon the horse seems to have been of the same nature. Though it was burnt also, it evidently made no struggle to escape, and its lips exhibited the same bloodless character.

The two men who escaped owed their safety primarily to a narrow passage, which acted efficiently as a barrier. Up to within twenty yards of the place where they were working the action of fire is apparent, the coal dust on the timbers having been converted into bright glistening coke. A puff of wind which extinguished one of their lamps made them suspicious that something was wrong, and they ran to the bottom of the pit. Neither seeing nor hearing anything suspicious, and no'

encountering any gas or afterdamp, they speedily returned and resumed their work. The blowing away of doors by the force of the explosion had evidently established such a circuit of air as carried the products of combustion away from them. One of them joined the first rescue party that descended the mine, and accompanied them in their explorations.

---

### TWO CASES OF INTUBATION IN ADULTS.

